# Discovering host-viral protein interactions in autophagy: A LIR discovery pipeline for identifying LC3-interacting region motifs in highly virulent viruses

**DOI:** 10.1371/journal.ppat.1014607

**Published:** 2026-09-11

**Authors:** Kaylee D. Petraccione, Haytham M. Wahba, Mohamed G. H. Ali, Timothy Stocker, Ivan Akhrymuk, Yossira Swese, Danuta Sastre, Andrew Silberfarb, Paul E. O’Maille, James G. Omichinski, Kylene Kehn-Hall

**Affiliations:** 1 Department of Biomedical Sciences and Pathobiology, Virginia-Maryland College of Veterinary Medicine, Virginia Polytechnic Institute and State University, Blacksburg, Virginia, United States of America; 2 Center for Emerging, Zoonotic, and Arthropod-borne Pathogens, Virginia Polytechnic Institute and State University, Blacksburg, Virginia, United States of America; 3 Department of Biochemistry and Molecular Medicine, Université de Montréal, Montréal, Quebec, Canada; 4 Department of Biochemistry, Faculty of Pharmacy, Beni-Suef University, Beni-Suef, Egypt; 5 Biosciences Division, S.R.I. International, Menlo Park, California, United States of America; 6 Artificial Intelligence Center, S.R.I. International, Menlo Park, California, United States of America; University of Wisconsin-Madison, UNITED STATES OF AMERICA

## Abstract

Hemorrhagic fever virus (HFV) infections are highly fatal, posing a significant global pandemic threat as they continue to emerge in new locations. HFVs and other highly virulent viruses (HVVs), such as Nipah virus, exploit host cell pathways, including the conserved host autophagy pathway, to promote viral replication. The Atg8/microtubule-associated protein 1 light chain 3 (LC3) proteins are necessary for autophagosome formation and maturation. Proteins interact with Atg8-family proteins through LC3-interacting region (LIR) motifs, which is a short linear motif (SLiM) found in intrinsically disordered regions of proteins. The presence of these motifs in viral components suggests they play a role in hijacking or evading the host autophagy pathway, yet the identification of functional LIR motifs in viral proteins remains limited. To address this gap, we developed the LIR Discovery Pipeline (LIR-DP) which integrates amino acid sequence pattern matching, with protein disorder prediction using IUPred3, and modeling with AlphaFold3. Using LIR-DP, we identified 43 putative LIR motifs in 166 proteins from 22 HVVs and predicted that 18 of these LIRs would be functional. *In vitro* and *in cellulo* laboratory experiments demonstrated that LIRs from the Marburg virus nucleoprotein, the Nipah virus phosphoprotein, the Ebola virus VP35, and the Rift Valley fever virus NSs protein bind to Atg8/LC3 family proteins. The aromatic amino acid in the first position of each LIR motif was found to be critical for these interactions. We provide evidence for the utility of the LIR-DP in identifying functional LIRs within HVV proteins which may provide valuable insight into the mechanism by which HVVs modulate the autophagy pathway during infection.

## Author summary

Hemorrhagic fever viruses (HFVs) are highly lethal pathogens that are rarely studied due to the high biosafety requirements. Viral proteins interact with host proteins to facilitate viral replication, and studying these interactions provide insights into pathways that could be therapeutically targeted. Many protein-protein interactions utilize short linear motifs (SLiMs) found in intrinsically disordered regions of proteins. Our work focused on the LC3-interacting region (LIR) motif, which enables interaction with the host autophagy Atg8/LC3 proteins. We integrated artificial intelligence (AI) with laboratory experiments to develop a novel LIR Discovery Pipeline (LIR-DP) which rapidly identifies potential functional LIRs in viral proteins. We validated potential functional LIRs in the following highly virulent viral proteins; Marburg virus nucleoprotein, Ebola virus VP35, and Nipah virus phosphoprotein; for which there is limited understanding of interactions with host proteins. Our results support the broader significance of the LIR-DP, suggesting its potential to also identify functional SLiMs in a wide range of proteins.

## Introduction

Hemorrhagic fever viruses (HFVs) are a group of highly pathogenic RNA viruses that pose a global health threat due to high mortality rates associated with their ability to induce viral hemorrhagic fever (VHF) [1, 2]. HFVs originate from a diverse group of viral families, including *Filoviridae*, *Flaviviridae*, *Hantaviridae*, *Nairoviridae*, *Peribunyaviridae*, *Phenuiviridae*, and *Arenaviridae*. In addition, Nipah virus (NiV) and Hendra virus (HeV) are high consequence pathogens within the *Paramyxoviridae* family that cause significant neurological and respiratory disease [3]. HFVs, together with NiV and HeV, are collectively referred to within as highly virulent viruses (HVVs). Case fatality rates of 50% and 60% for Marburg virus (MARV) and Ebola virus (EBOV), respectively, demonstrate the devastating potential of VHF infections [4–6]. Importantly, several HVVs are classified as select agents by the CDC since, in addition to posing a serious public health risk, they have the potential to be utilized as bioweapons. Due to their significant risk to public health and the lack of effective vaccines and therapeutics, HVVs remain a critical focus for pandemic preparedness [7].

The lack of countermeasures for these viruses, is in part due to a limited understanding as to how HVVs interact with host cell pathways during infections. One host cell process that has been shown to be targeted by viruses, including several HVVs, during infection is macroautophagy (here after referred to as autophagy) [8–11]. Autophagy is a process whereby cellular materials are degraded and recycled to help maintain cellular homeostasis, and it is a critical component of the antiviral response in many host cells. During autophagy, cellular cargoes are targeted to autophagosomes, which later fuse with the lysosome where they are ultimately degraded. In response to many different types of viral infections, autophagy is activated in the host cell, which increases the host cell’s ability to engulf and degrade viral components and thereby limit viral replication [12–14]. Conversely viral infection may trigger autophagy to facilitate viral replication. For example, cells infected with the Junin virus (JUNV) exhibited increased autophagy, and inhibition of autophagy through either rapamycin treatment or depletion of ATG5 significantly reduced viral replication [15]. Furthermore, multiple positive-sense RNA viruses induce autophagy and may co-opt autophagosomes as sites of viral replication [9]. This underscores the dual role of autophagy in either promoting or restricting viral infection depending on the viral context.

Atg8/LC3 proteins regulate autophagy by controlling the growth and maturation of autophagosomes as well as their fusion to lysosomes [16, 17]. They also play roles beyond autophagy through Atg8ylation, which is the process of conjugation of Atg8/LC3 proteins to membranes including non-autophagosomes [18, 19]. In humans, there are six Atg8/LC3-family members, and they include LC3A, LC3B, LC3C, GABARAP, GABARAPL1, and GABARAPL2. These proteins carry out many of their functions due to their capacity to interact with other autophagy factors that contain a short motif referred to as the LC3-interacting region (LIR) motif [20, 21]. Host proteins that have been shown to contain functional LIR motifs include several members of the basal autophagy machinery, Rab GTPase-activating proteins (GAPS), and autophagy specific cargo receptors [22]. The core of the LIR motif consists of just four amino acids with the canonical sequence W/F/Y-x-x-L/V/I, where x represents any amino acids (aa), with the first aromatic residue and the last hydrophobic residue being crucial for binding to Atg8/LC3 proteins [21]. However, select interactions with the individual human Atg8/LC3-family proteins have been shown to be also regulated by amino acids that flank both the N- and C-terminal sides of the core LIR motif [23]. Specifically, negatively charged amino acids (Glu/Asp) or phosphorylated residues (Ser/Thr) to the N-terminal of the LIR motif often facilitate interactions [20]. Given that a LIR is only four amino acids in length, one important feature for identifying a functional LIR motif in a protein is that they are usually located in intrinsically disordered regions (IDRs) and this is consistent with the LIR motif being classified as a short-linear interacting motif (SLiM) [24]. More generally, SLiMs are segments (typically less than 10 amino acids in length) typically found in IDRs of proteins that play critical roles in coordinating cellular processes often through their participation in protein-protein interactions. The inherent flexibility of SLiMs allows them to transiently associate with multiple partners to form cellular complexes, and these interactions can be easily regulated by post-translational modifications. This makes SLiMs ideal for participating in interactions that regulate signaling, cellular localization, protein degradation, and proteolytic cleavage, key molecular events that drive cellular pathways (e.g., immunity, homeostasis, stress responses) that are rapidly evolvable, as reviewed [25–27].

Since many viral proteins have been shown to use SLiMs to interact with host proteins as part of their functions [28], it is not surprising that LIR motifs have been identified in viral proteins. Viruses typically have limited coding capacity, and the short sequences of SLiMs provide an easy means to mediate protein-protein interactions. In addition, these sequences can be readily altered through evolution, making them advantageous from an evolutionary perspective [29]. The importance of a functional LIR in a viral protein was first documented in the influenza A virus, where it was shown that the viral M2 protein interacts with Atg8/LC3 proteins via its LIR motif to promote viral budding and stability [30]. An example of a functional LIR motif in a HVV protein was recently identified in the non-structural small (NSs) protein of the Rift Valley fever virus (RVFV). The LIR motif is located at the very C-terminus of the NSs protein, and it was shown to be required by the RVFV to inhibit autophagy through interactions with several different human Atg8/LC3 proteins, and this interaction helps promote viral replication in infected cells [10].

The importance of interactions involving LIR motifs has motivated the development of specialized databases, for example, the iLIR database [31], which has compiled predictions of LIR motifs in viral and host proteomes. Further, the iLIR database offers sequence searches using the four or six amino acid consensus sequence for LIR motifs, the WxxL or xLIR, respectively. While this resource has been a widely used tool by the autophagy community, the identification of functional LIR motifs in viral proteins has not been extensively investigated. This is in part due to the fact that the consensus sequence for an LIR motif is only four amino acids. Thus, there are many examples of LIR patterns in both host and viral proteins, but many of these sequences serve as key structural elements of protein domains and therefore are not available to function as an LIR motif. In addition, researchers are beginning to recognize the impact of flanking residues outside of the consensus motif for providing context for LIR-mediated interactions. To address these issues, we developed The LIR Discovery Pipeline (LIR-DP). LIR-DP integrates LIR sequence pattern matching, including upstream and downstream flanking residues beyond the four or six amino acid motif, with prediction of protein disorder with IUPred3, and structural modeling of LIR-LC3 complexes using AlphaFold3 (AF3). Importantly LIR-DP excludes LIR motifs within protein regions required to maintain the structural integrity of a folded protein domain. To test the validity of LIR-DP, we examined 166 proteins from 22 different HVVs and we predicted the presence of 18 potential functional LIR motifs. Several of these predicted functional LIR motifs were then examined for their ability to interact with Atg8/LC3-family proteins both *in vitro* using isothermal titration calorimetry (ITC) and *in cellulo* experiments. The results demonstrated that the Marburg virus (MARV) nucleoprotein (NP), the Ebola virus (EBOV) VP35, and the Nipah virus (NiV) phosphoprotein (P) contain functional LIR motifs. The present study demonstrates the utility of the LIR-DP to identify functioning LIR motifs in HVVs and suggests that this pipeline can be used to identify functional LIR motifs in other host and viral proteins. In addition, this versatile pipeline can be easily adapted to identify other types of functional SLiMs in host and viral proteins.

## Results

### The LIR discovery pipeline: identification of HVV proteins containing LIR motifs

**Fig 1:**
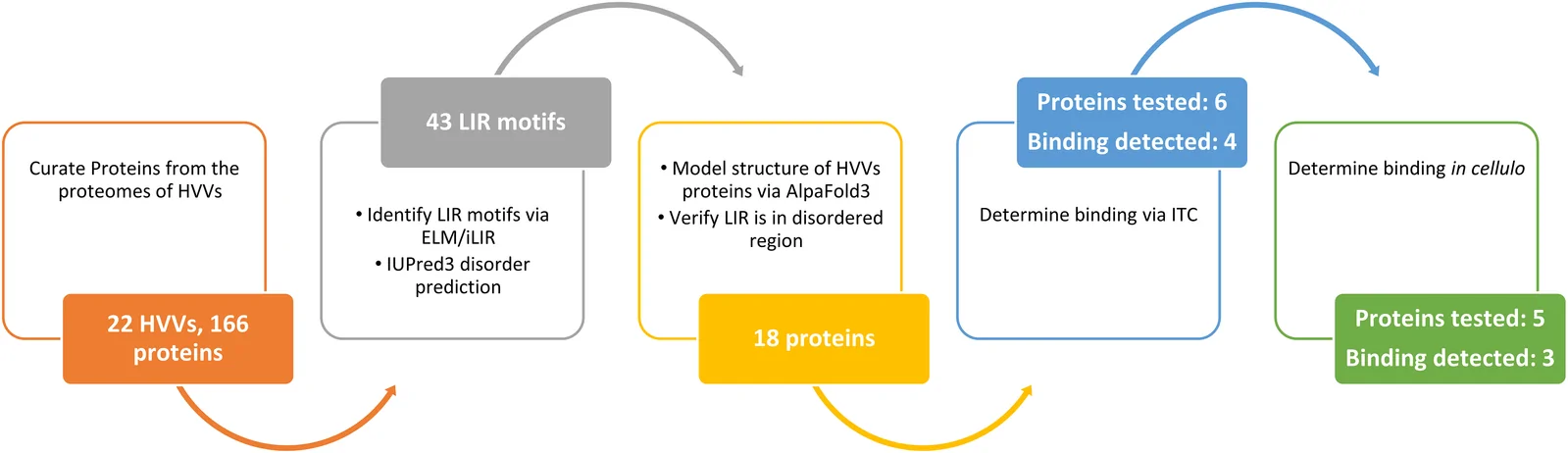
LIR discovery pipeline. A total of 166 viral proteins from 22 highly virulent viruses (HVVs) were curated and screened for LC3-interacting region (LIR) motifs using both ELM and iLIR. The primary pattern used was LIG_LIR_GEN_1 (http://elm.eu.org/elms/LIG_LIR_Gen_1) which considers up to 2 residues before and 8 residues after the core 4 residue sequence. All LIR motifs were further analyzed to select only those that were in disordered protein regions based on IUPred3 predictions, resulting in the identification of 43 candidate LIR motifs. Structural models of the full-length proteins containing the candidate LIR motifs were generated using AlphaFold3. Based on structural modeling and additional pipeline criteria, 18 proteins were classified as high-confidence for the presence of a functional LIR motif of which 3 of the LIR motifs were identical (thus 16 different LIR motifs). Peptide fragments (16 amino acids long) containing the 16 different candidate LIR motifs were modeled in complex with both LC3A and GABARAP using AlphaFold3 and the strength of the protein-protein interfaces were ranked using FoldX. Six of the candidate LIR motifs were tested *in vitro* and five candidate LIR motifs were tested *in cellulo* for binding to GABARAP and/or LC3A.

An artificial intelligence/machine learning (AI/ML)-driven LIR discovery pipeline was developed to identify and analyze LIR motifs in HVV proteins (Fig 1). First, we generated a list of 166 non-redundant viral proteins encoded by 22 HVVs (S1 Table), which were then analyzed using both the eukaryotic linear motif (ELM) and iLIR databases to identify putative LIR motifs within the protein sequences [31, 32]. Since LIR motifs are considered to be generally located only in disordered regions of proteins [24], the LIR motifs identified using ELM and iLIR were further analyzed using IUPred3 [33] to estimate the relative disorder score of the region they were located in the protein sequence. Those regions with an average disorder score above 30 over the residues that match the LIR motif were moved to the next step in the pipeline. This low cutoff is for computational efficiency to limit the number of predicted structures that are calculated. Its presence produces a negligible increase in false negatives that we can’t detect in our current sample. Subsequent analysis used in-silico binding analysis for more detailed rejection. This initial phase of the analysis led to the identification of 43 of the 166 proteins containing putative LIR motifs to be considered in disordered regions (S2 Table). Next, we used AlphaFold3 to generate a full-length structural model of each of the 43 HVV proteins containing the putative LIR motifs. These models were used to further validate that the LIR was likely to be in a disordered region of the protein. The predicted local distance difference test (pLDDT) was used as an independent indicator of disorder with a pLDDT below 50 confirming a prediction of the LIR motif being present in a disordered region of the protein. Based on the AlphaFold3 predictions, 18 of the 43 proteins (Table 1) were predicted to have an LIR motif within a region with a pLDDT score below 50. These 18 proteins were moved on to the next step in the pipeline, whereas the remaining 25 proteins that were found to contain LIR motifs within more structured protein regions (pLDDT score above 50), were excluded from subsequent steps of analysis. Representative examples of pLDDT predictions are shown in S1 Fig, where MARV nucleoprotein (NP) (S1A and S1B Fig) showed a favorable pLDDT score below 50 and Lassa virus (LASV) NP (S1C and S1D Fig), had a pLDDT score above 50.

**Table 1:** Final list of 18 putative LIR-containing proteins from HVVs. Out of 166 HVV proteins analyzed using the LIR Discovery Pipeline, 18 proteins were predicted to contain functional LIR motifs capable of binding to Atg8/LC3- proteins.

Number	Virus	Protein	LIR Sequence	LIR Start Amino Acid	LIR Stop Amino Acid	Uniprot ID
1	CCHFV	RNA-directed RNA polymerase L	YEDL	191	194	Q6TQR6
2	EBOV	RNA-directed RNA polymerase L	FQSF	1714	1717	Q05318
3	MARV	RNA-directed RNA polymerase L	FQIL	1797	1800	P31352
4	NiV	RNA-directed RNA polymerase	FNKV	640	643	A0A514MHJ1
5	PUUV	RNA-directed RNA polymerase	FTTV	1953	1956	P0C760
6	DENV	Capsid protein (CP)	FNML	12	15	P17763_10
7	GTOV	Z	YSSL	10	13	A2RQH3
8	EBOV	Glycoprotein (GP)	FTVV	307	310	Q05320
9	EBOV	Polymerase cofactor VP35	FEEV	82	85	Q05127
10	RVFV	Non-structural protein S (NSs)	FVEV	261	264	A2SZY8
11	DBV	Non-structural protein (NSs)	WPAI	283	287	A0A0D6A9A2
12	SEOV	Nucleoprotein (NP)	YEDV	164	167	Q808S4
13	DOBV	Nucleoprotein (NP)	FEDV	164	167	S5MI15
14	PUUV	Nucleoprotein (NP)	FEDI	164	167	P27313
15	MARV	Nucleoprotein (NP)	FVDL	456	459	P27588
16	NiV	Phosphoprotein (P)	FEQI	55	58	A0A857UX52
17	NiV	W protein	FEQI	55	58	A0A857UYC2
18	NiV	Non-structural protein V	FEQI	55	58	A0A857UX58

### HVV proteins interact with LC3A *in silico* via LIR motifs

**Fig 2:**
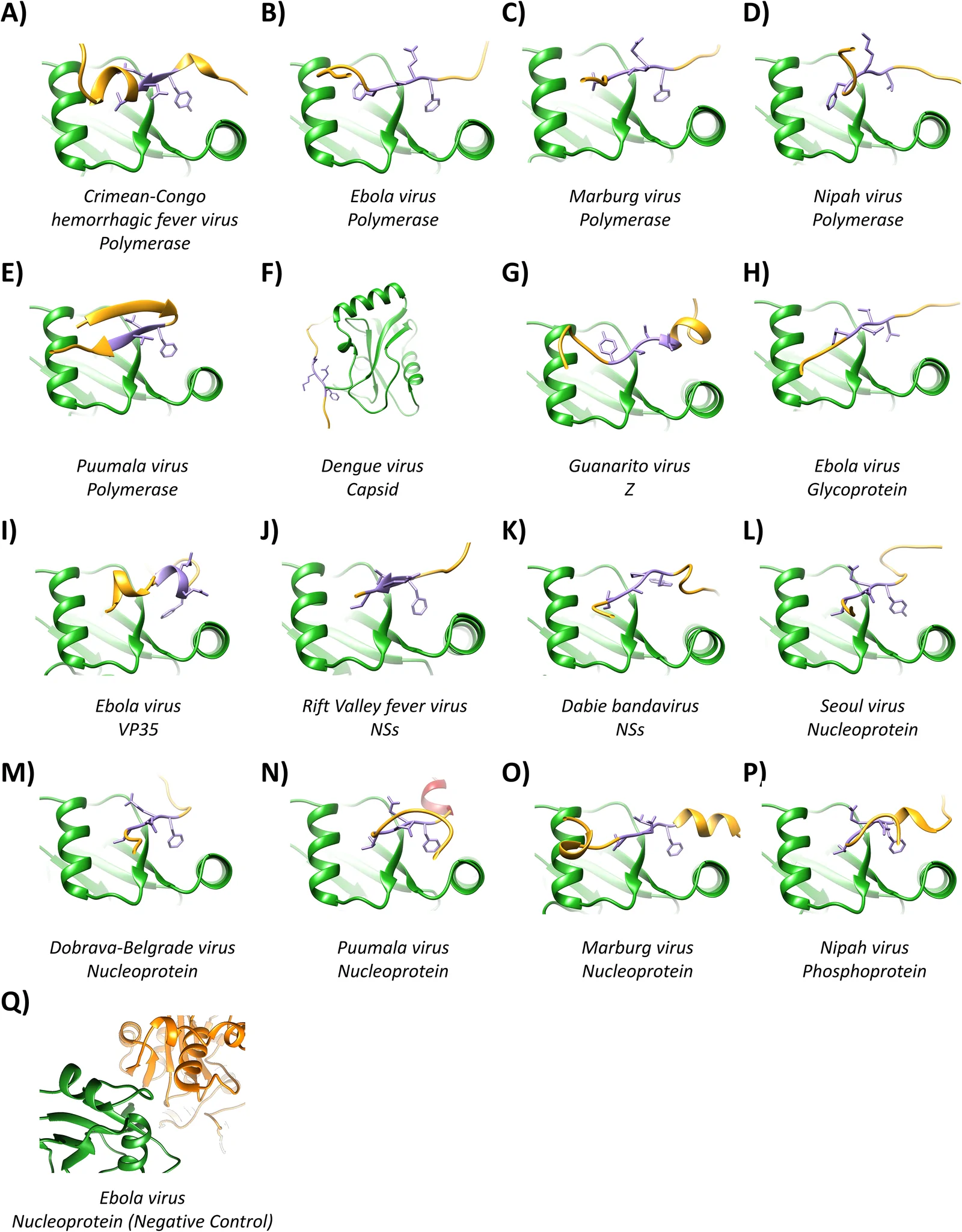
HVV proteins interact with LC3A in silico via LIR motifs. Representative structural models of HVV LIR motifs (purple) in complex with LC3A (green) proteins, as calculated via AlphaFold3 and FoldX (Materials and Methods).

In the next step, the 16 out of the 18 putative HVV protein candidates from Table 1 were modeled in complex with Atg8/LC3-family members using AlphaFold3 (Fig 2). The NiV P/V/W proteins are all produced from the P gene through RNA editing and thus contain the identical putative LIR motif due to a common N-terminal domain [34]. Given this, the NiV P was analyzed as a representative of these 3 proteins. In many cases, the AlphaFold3 predictions failed to generate an accurate model of the complex with the full-length viral proteins and this is consistent with the fact that it has been documented that AlphaFold3 is still not well suited for identifying a preferred binding interface for complexes when modeling larger proteins. Despite its power, AlphaFold3 still has considerable limitations in modeling the structures of complexes involving disordered protein regions [35]. To compensate for this deficiency, we added an additional step to our AI-based protocol. In this additional step, AlphaFold3 was used to model complexes with shorter segments of the proteins that contain the LIR motifs of interest. The minimal segment length used was 16 amino acids, with the LIR located at the center of the sequence, but we also tested fragments of 20, 30 and 50 amino acids in length with AlphaFold3. These lengths all capture the core LIR motif as well as nearby residues allowing AlphaFold to correctly account for non-SLiM effects, such as protein charge / isoelectric point (pI in S3 Table). These models were then evaluated based on predicted binding energy, intrinsic disorder of the unbound fragment, and the spatial interaction between the viral and host proteins utilizing FoldX (Table 2). Binding energy on the AlphaFold3 structure was measured as the FoldX energy of the full structure minus the energy of its components when separated. This is slightly lower than the actual binding energy due to the separate components relaxing, however, it is more consistent. Specificity was not directly measured, but rather implicitly measured as a difference in binding energy across the range of structures considered. To evaluate the consistency and accuracy of AlphaFold3/FoldX model outputs, structures of complexes were analyzed by UCSF Chimera [36] Specifically, all models of HVV peptides containing LIR motifs in complex with LC3A were superimposed using Matchmaker with the best-aligning pair of chains (i.e., LC3A). The extent to which peptide segments with the HVV LIR motifs bound to LC3A in a canonical manner (e.g., positioning of consensus residues and polarity) was assessed by visual comparison with reference crystal structures of LIR motif bound to representative Atg8/LC3-family members (e.g., PDBID 8T2N; Crystal structure of GABARAP in complex with the LIR of NSs3). Representative low energy complexes of the HVV viral proteins with LC3A are shown in Fig 2. EBOV NP, which does not contain a putative LIR, was modeled with LC3A as a negative control and demonstrated no interaction with LC3A (Fig 2Q). In contrast, the 16 HVV proteins with putative LIRs all demonstrated binding with LC3A (Fig 2A-2P).

**Table 2:** Biophysical parameters of in silico interactions between HVV LIR-containing peptides and LC3A.

Virus	Protein	Binding Energy	Δ Multimer ASA
CCHFV	RNA-directed RNA polymerase L	-23.907	0.306
EBOV	RNA-directed RNA polymerase L	-17.194	0.242
MARV	RNA-directed RNA polymerase L	-25.077	0.336
NiV	RNA-directed RNA polymerase L	-18.696	0.301
PUUV	RNA-directed RNA polymerase L	-21.838	0.276
DENV	Capsid protein (CP)	-21.652	0.291
GTOV	Z	-15.652	0.285
EBOV	Envelope glycoprotein (GP)	-18.967	0.311
EBOV	Polymerase cofactor VP35	-18.127	0.292
RVFV	Non-structural protein small (NSs)	-28.43	0.444
DBV	Non-structural protein	-13.820	0.256
SEOV	Nucleoprotein (NP)	-24.338	0.279
DOBV	Nucleoprotein (NP)	-22.606	0.269
PUUV	Nucleoprotein (NP)	-19.329	0.281
MARV	Nucleoprotein (NP)	-19.993	0.353
NiV	Phosphoprotein (P)	-17.345	0.335

Using this pipeline, a list of 18 proteins that contain 16 different putative LIR motifs (See note below) was created that includes the already characterized LIR motif of the Rift Valley fever virus (RVFV) NSs as well as 5 RNA directed RNA polymerases (Marburg virus (MARV), Ebola virus (EBOV), Crimean Congo hemorrhagic fever virus (CCHFV), Nipah virus (NiV), and Puumala virus (PUUV)), 4 nucleoproteins (MARV, Seoul virus (SEOV), PUUV and Dobrava-Belgrade virus (DOBV)), dengue virus (DENV) capsid protein (CP), EBOV glycoprotein, EBOV VP35 protein, Guanarito virus (GTOV) Z, Dabie bandavirus (DBV) NSs, and the phosphoprotein (P), non-structural V protein, and W protein of NiV (Table 1). (Note: The NiV P/V/W proteins contain the identical putative LIR motif due to a common N-terminal domain [34]. This LIR is referred to as NiV P/V/W when only a peptide containing the LIR motif is used for experiments). This initial modeling and ranking laid the foundation for priority candidate LIR motifs for further characterization through biochemical and cell-based assays as described below. Given the large size of viral polymerases, these proteins were deprioritized for validation studies.

### Validation of the putative LIR motifs in HVVs binding to Atg8/LC3-family proteins *in vitro*

The Atg8/LC3 family of proteins can be further divided into subfamilies with LC3A, LC3B, and LC3C belonging to the LC3 subfamily and GABARAP, GABARAPL1 and GABARAPL2 belonging to the GABARAP subfamily [21]. To validate the interaction of the LIR motifs identified using the LIR Discovery Pipeline (LIR-DP) we performed ITC experiments with at least one member from each subfamily. GABARAP, LC3A, and LC3B proteins were expressed and purified, and their binding to select peptides containing the putative LIR motifs from HVV proteins determined using isothermal titration calorimetry (ITC) experiments (Table 3). ITC experiments were performed with five peptides containing putative LIR motifs identified by the LIR-DP: MARV NP-LIR, NiV P/V/W-LIR, EBOV VP35-LIR, GTOV Z-LIR, and DENV CP-LIR. As a positive control, the RVFV NSs-LIR, which we have previously characterized [10], was included in the analysis. The RVFV NSs-LIR peptide had binding affinities of 0.24 ± 0.05 μM for LC3A, 0.60 ± 0.03 μM for LC3B, and 0.43 ± 0.03 μM for GABARAP and served as a benchmark for comparison with the other peptides. The ITC experiments revealed that MARV NP-LIR peptide bound to LC3A, LC3B, or GABARAP with low micromolar affinity. The K_D_ values for MARV NP-LIR were 2.2 ± 0.4 μM for LC3A, 0.60 ± 0.03 μM for LC3B, and 1.0 ± 0.1 μM for GABARAP. The NiV P/V/W-LIR and EBOV VP35-LIR peptides bound to both GABARAP, LC3A, and/or LC3B but with affinities in the low to high micromolar range. The K_D_ value for NiV P/V/W-LIR was 6.9 ± 0.1 μM for GABARAP, 9.7 ± 0.01 μM for LC3A, and 33.0 ± 0.14 μM for LC3B. The K_D_ value for EBOV VP35-LIR was 13 ± 2 μM for GABARAP and 390 ± 10 μM for LC3A. Notably, both NiV P/V/W-LIR and EBOV VP35-LIR bound with higher affinities to GABARAP as compared to LC3A. For the GTOV Z-LIR and DENV CP-LIR peptides there was no heat detected, and thus no evidence for binding between these peptides and either LC3A or GABARAP under the experimental conditions used for the ITC studies. However, analysis of the sequences of the DENV CP-LIR and GTOV Z-LIR peptides indicates that they both possess very high isoelectric points (pIs greater than 9) (S3 Table). This is of note because it is well established that functional LIR motifs are often flanked by negatively charged amino acids, including phosphorylated amino acids, that augment the binding affinity to Atg8/LC3-family proteins [22, 37]. This is consistent with the LIR-binding regions of Atg8/LC3-family proteins being highly electropositive. These ITC results with the DENV CP-LIR and GTOV Z-LIR peptides are also supported by experiments in cells with the full-length DENV CP and GTOV Z proteins, where no co-immunoprecipitation was observed with LC3 (S2 Fig). These results demonstrate that RVFV NSs-LIR and MARV NP-LIR peptides both interact with a low micromolar to high nanomolar affinity to Atg8/LC3-family members via ITC. In contrast, both NiV P/V/W-LIR and EBOV VP35-LIR interact with a low micromolar to high micromolar affinity to Atg8/LC3-family members via ITC.

**Table 3:** ITC experiments of putative LIR motifs binding to Atg8/LC3-family proteins.

Peptide	GABARAP	LC3A	LC3B
RVFV NSs-LIR	0.43 ± .03 μM	0.24 ± 0.05 μM	0.60 ± 0.03 μM
MARV NP-LIR	1.0 ± 0.1 μM	2.2 ± 0.4 μM	3.0 ± 0.5 μ ε34 5M
NiV P/V/W-LIR	6.9 ± 1.0 μM	9.7 ± 0.1 μM	33 ± 14 μM
EBOV VP35-LIR	13 ± 2.0 μM	390 ± 30 μM	-------------
GTOV Z-LIR	n.d	n.d	n.d.
DENV CP-LIR	n.d	n.d	n.d.

n.d-no heat detected

### Validation of NiV P interacting with GABARAP *in cellulo* through its putative LIR motif

**Fig 3:**
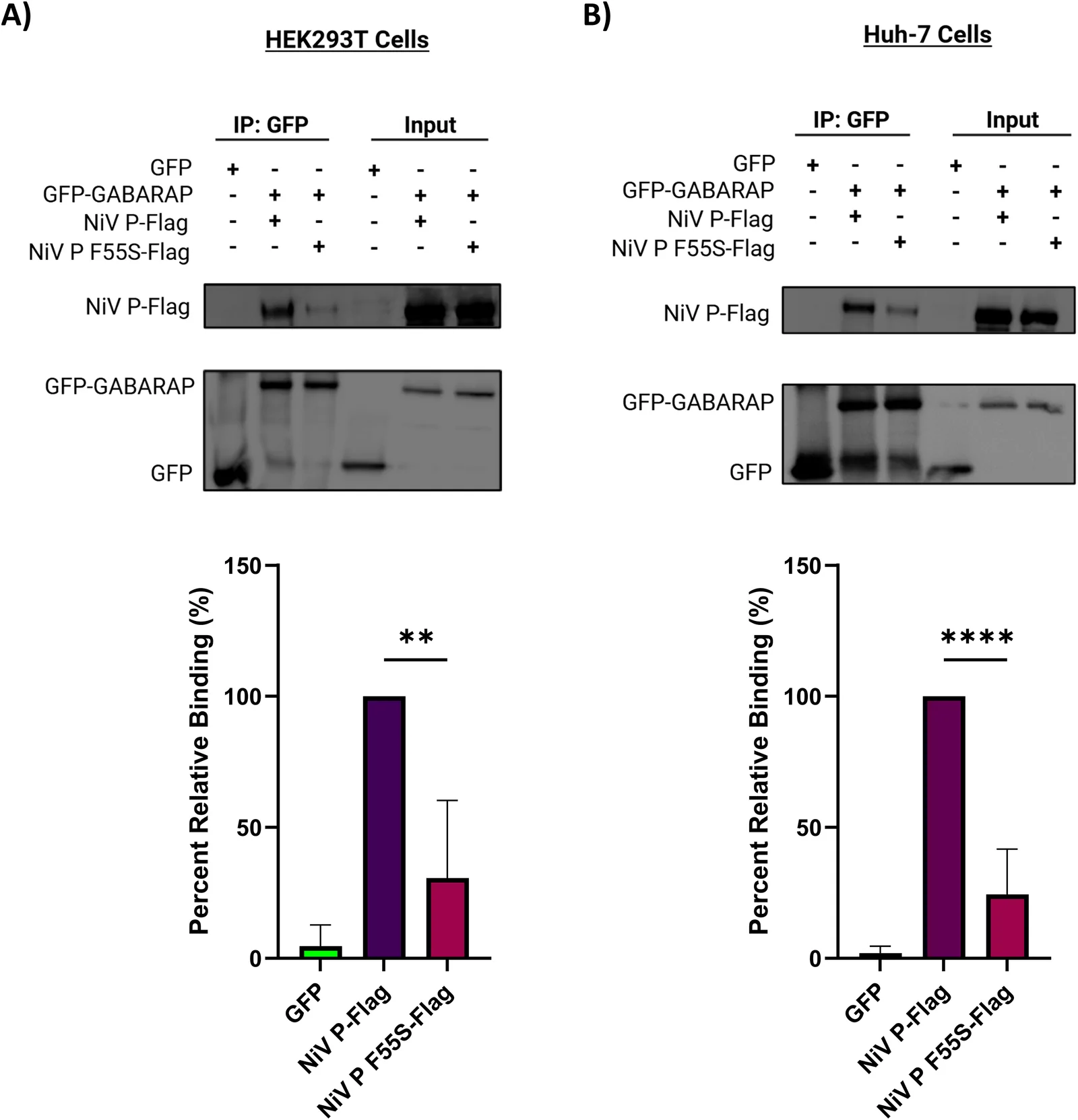
NiV P interacts with GABARAP via the predicted LIR motif *in cellulo.* A) HEK293T cells were co-transfected with GABARAP-GFP and the indicated viral plasmids and collected 24 hpt. Coimmunoprecipitation was performed with anti-GFP antibodies and western blot performed with Flag and GFP antibodies. Quantitation of 3 biological replicates from independent transfection experiments is shown. B) Same as panel B but with Huh7 cells. ****p-value <0.0001.

To determine if the full-length NiV P interacts with Atg8/LC3-family proteins via its putative LIR motif (^55^FEQI^58^) in human cells, co-immunoprecipitation assays were performed in both HEK293T and Huh-7 cells transfected with a plasmid expressing NiV P 3XFlag, in which a triple tandem Flag tag is incorporated at the C-terminus of the NiV P protein (NiV P-Flag). Initial experiments were performed to assess the interaction between NiV P and LC3 due to LC3 antibodies being readily available and optimized for coimmunoprecipitation experiments (Note the LC3 antibody recognizes both LC3A and LC3B and for this reason is referred to as just LC3). Twenty-four hours post transfection (24 hpt), cells were lysed and subjected to co-immunoprecipitation with an anti-LC3 antibody, followed by western blot analysis with anti-LC3 and anti-Flag antibodies. No detectable interaction between NiV P and LC3 was observed under these experimental conditions (S3 Fig). Thus, given that the ITC results (Table 3) indicated stronger binding between the NiV P/V/W-LIR and GABARAP *in vitro*, experiments were next performed to test the ability of NiV P to interact with GABARAP in *cellulo*. To determine whether the LIR motif was critical for NiV P-GABARAP interaction, a plasmid encoding an NiV P F55S variant was also tested, which contains an amino acid substitution of phenylamine to serine at the first position of the LIR motif (^55^FEQI^58^). HEK293T cells and Huh7 cells were transfected with plasmids expressing GFP (as a negative control), the NiV P-Flag and GFP-GABARAP, or a NiV P F55S-Flag variant and GFP-GABARAP. Twenty-four hpt, cells were lysed and subjected to co-immunoprecipitation with an anti-GFP antibody, and then western blot analysis was performed using anti-GFP and anti-Flag antibodies. The analysis of the co-immunoprecipitation samples indicated that wild-type NiV P interacts with GABARAP in both HEK293T (Fig 3A) and Huh-7 (Fig 3B) cells, but the NiV P F55S variant displayed significantly reduced binding to GABARAP in both HEK293T (Fig 3A) and Huh-7 (Fig 3B) cells. These results are consistent with the LIR-DP prediction that the NiV P protein contains a functional LIR motif (^55^FEQI^58^) and like other functional LIR motifs the aromatic residue in position one (F55) is important for its interaction with Atg8/LC3-family proteins in human cells.

### Validation of EBOV VP35 interacting with LC3 *in cellulo* via its putative LIR motif

**Fig 4:**
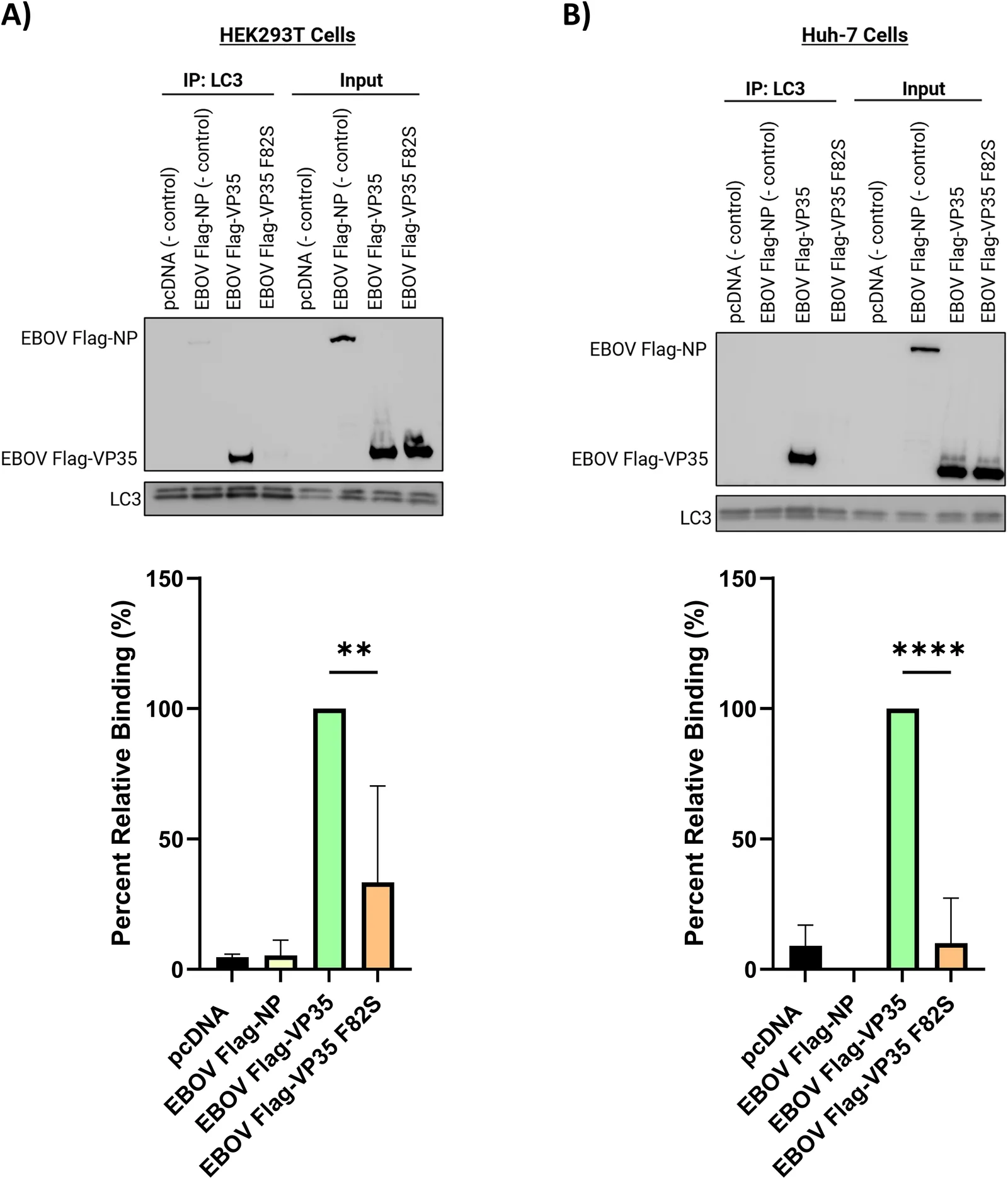
EBOV VP35 interacts with LC3 via the predicted LIR motif *in cellulo.* A) HEK293T cells were transfected with the indicated plasmids and collected 24 hpt. Coimmunoprecipitation was performed with anti-LC3 antibodies and western blot performed with Flag and LC3 antibodies. Quantitation of 3 biological replicates from independent transfection experiments is shown. B) Same as panel B but with Huh7 cells. ****p-value <0.0001.

Next, we tested whether the full-length EBOV VP35 protein can also interact with either GABARAP or LC3 via its putative LIR motif (^82^FEEV^85^) in cells as predicted with LIR-DP. To examine the interaction between EBOV VP35 and LC3, both HEK293T and Huh-7 cells were transfected with plasmids expressing either the EBOV VP35 protein with an N-terminal Flag (EBOV Flag-VP35), an EBOV Flag-VP35 F82S variant, or an EBOV Flag-NP negative control. As with the NiV P protein, the substitution of phenylalanine for serine (F82S) in the first position of the LIR motif was included to verify that the putative LIR motif is important for a functional interaction between EBOV VP35 and LC3 in human cells. Twenty-four 24 hpt, cells were lysed and subjected to co-immunoprecipitation using an anti-LC3 antibody, followed by western blot analysis with anti-LC3 and anti-Flag antibodies. The analysis of the co-immunoprecipitation samples indicated that EBOV VP35 interacts with LC3 in both HEK293T (Fig 4A) and Huh-7 (Fig 4B) cells. In contrast, the interaction with the EBOV VP35 variant containing the F82S substitution showed a significant reduction in binding to LC3 in comparison to what was seen with the WT protein. Again, this demonstrated that the EBOV VP35 protein contains a functional LIR motif (^82^FEEV^85^) as predicted by LIR-DP, and that the aromatic residue in position one (F82) is crucial for its interaction with Atg8/LC3-family proteins in human cells (Fig 4).

To examine the potential interaction between EBOV VP35 and GABARAP in cells, Huh7 cells were transfected with plasmids expressing GFP (as a negative control), EBOV Flag-NP (as an additional negative control), EBOV Flag-VP35, an EBOV Flag-VP35 F82S variant and/or GFP-GABARAP. Twenty-four hpt, cells were lysed and subjected to co-immunoprecipitation with an anti-GFP antibody, and then western blot analysis was performed using anti-GFP and anti-Flag antibodies. No interaction between EBOV VP35 and GABARAP was observed in Huh7 cells (S4 Fig).

### Validation of MARV NP interacting with LC3 *in cellulo* via its putative LIR motif

**Fig 5:**
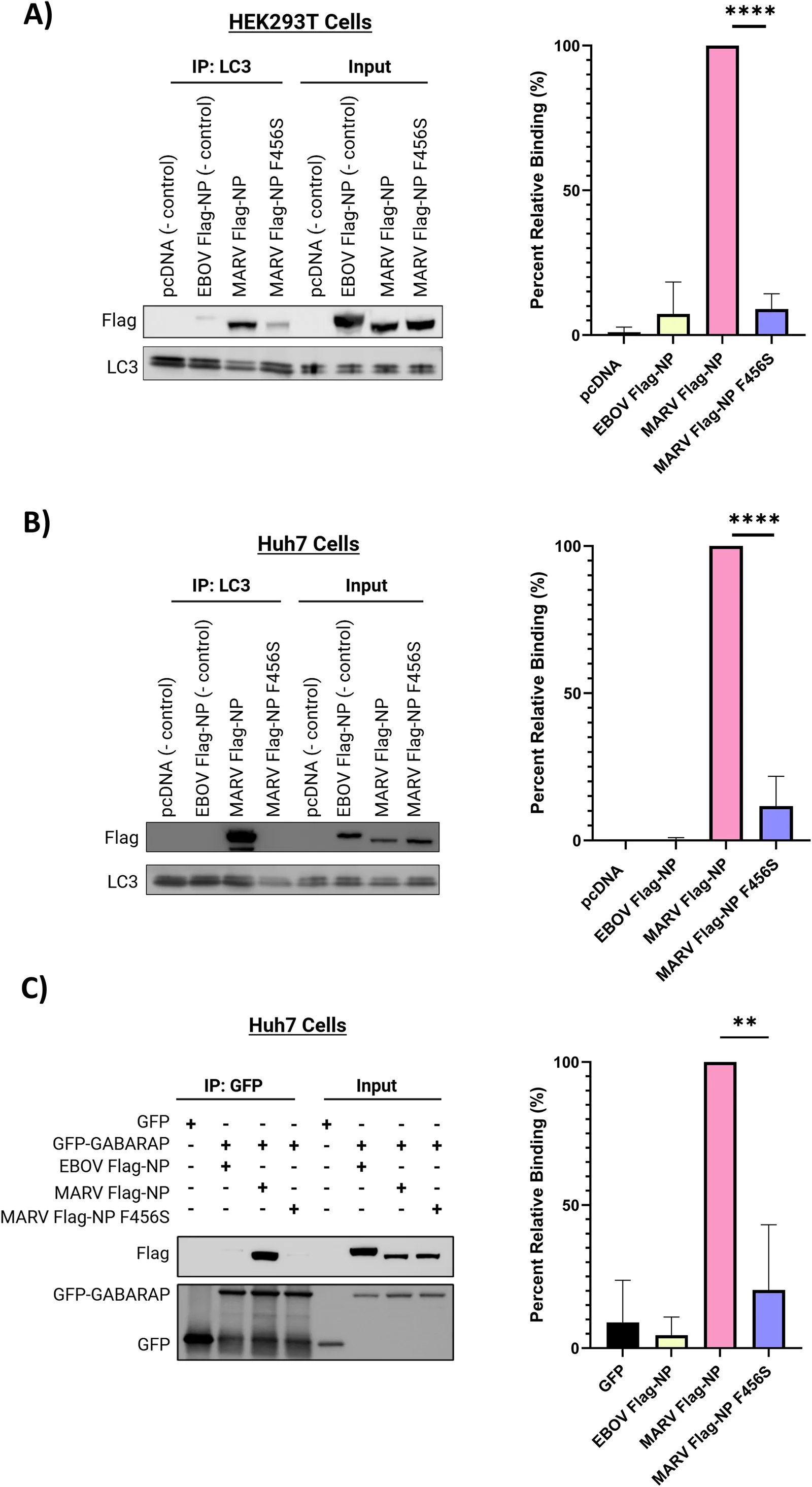
MARV NP interacts with LC3 and GABARAP via the predicted LIR motif *in cellulo.* A) HEK293T cells were transfected with the indicated plasmids and collected 24 hpt. Coimmunoprecipitation was performed with anti-LC3 antibodies and western blot performed with Flag and LC3 antibodies. The right panel displays quantitation of 3 biological replicates from independent transfection experiments. B) Same as panel A but with Huh7 cells. C) Huh7 cells were transfected with the indicated plasmids and collected 24 hpt. Coimmunoprecipitation was performed with anti-GFP antibodies and western blot performed with Flag and GFP antibodies. The right panel displays quantitation of 3 biological replicates from independent transfection experiments, except for EBOV Flag-NP which includes quantitation from 2 biological replicates from independent experiments. **p-value <0.01, ****p-value <0.0001.

To verify that the full-length MARV NP interacts with LC3 via the putative LIR motif (^456^FVDL^459^) in cells, HEK293T and Huh-7 cells were transfected with plasmids expressing either the MARV Flag-NP, a MARV Flag-NP F456S variant, or the negative control EBOV Flag-NP. As with the NiV P and EBOV VP35, a variant with a substitution of F456 for serine (F456S) in the LIR motif was included to verify that the putative LIR motif predicted by the LIR-DP is important for a functional interaction between MARV NP and LC3 in cells. Twenty-four hpt, cells were lysed and subjected to co-immunoprecipitation with the anti-LC3 antibody, followed by western blot analysis with anti-LC3 and anti-Flag antibodies. Analysis of the co-immunoprecipitation samples indicated that MARV NP interacts with LC3 in *cellulo* in both HEK293T (Fig 5A) and Huh-7 (Fig 5B) cells. In contrast, the variant MARV NP with the F456S substitution showed significantly less binding to LC3.

To examine the potential interaction between MARV NP and GABARAP in cells, Huh7 cells were transfected with plasmids expressing GFP (as a negative control), EBOV Flag-NP (as an additional negative control), MARV Flag-NP, a MARV Flag-NP F456S variant and/or GFP-GABARAP. Twenty-four hpt, cells were lysed and subjected to co-immunoprecipitation with an anti-GFP antibody, and then western blot analysis was performed using anti-GFP and anti-Flag antibodies. The analysis of the co-immunoprecipitation samples indicated that wild-type MARV NP interacted with GABARAP in Huh-7 cells (Fig 5C), but the MARV NP F456S variant displayed significantly reduced binding to GABARAP. These results, coupled with the data shown in Fig 5A and Fig 5B, demonstrate the importance of the LIR motif (^456^FVDL^459^) for the interaction of MARV NP with LC3 and GABARAP in human cells (Fig 5).

### Structures of MARV NP-LIR in complex with Atg8/LC3-family proteins

**Fig 6:**
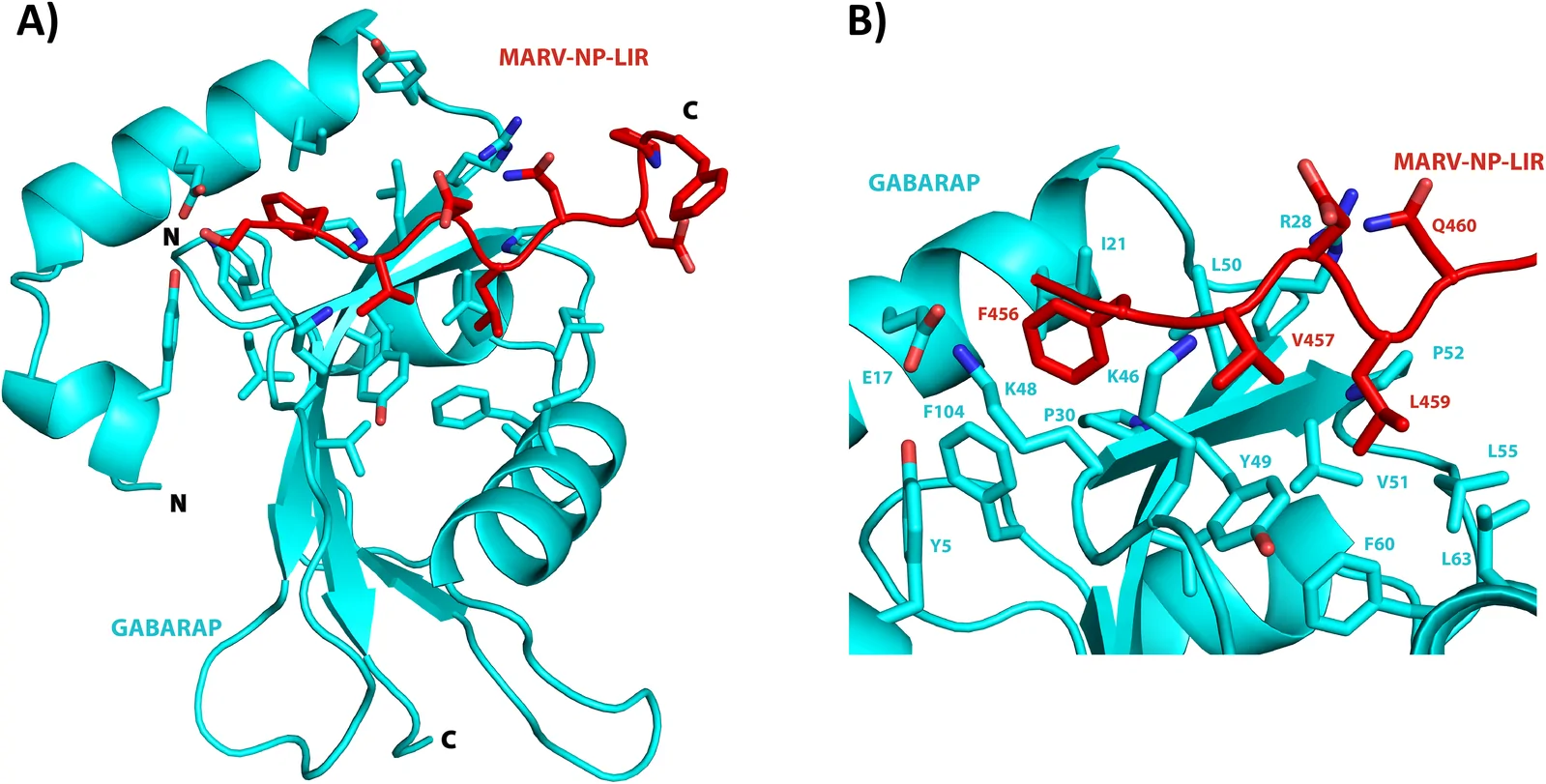
Crystal structure of MARV NP-LIR in complex with GABARAP. A) Cartoon representation of the crystal structure of the MARV NP-LIR in complex with GABARAP. The side chains of MARV NP-LIR (firebrick red) are shown when complexed with GABARAP (cyan). B) Closeup of A highlighting the binding of F456 and L459 of the MARV NP-LIR in HP1and HP2 of GABARAP, respectively.

Among the putative LIR motifs identified with the LIR-DP that were tested *in vitro* and *in cellulo*, the MARV NP-LIR displayed the highest binding affinity for both GABARAP and LC3A *in vitro* by ITC (Table 3). Given this, attempts were made to obtain crystal structures of complexes between the MARV NP-LIR and both GABARAP and LC3A. All screening attempts to generate co-crystals of the complexes were unsuccessful, but crystals of a complex were obtained using a fusion protein consisting of the MARV NP-LIR attached to the N-terminal of GABARAP (residues 454–463 of MARV NP fused to GABARAP with a two glycine residue separation) that diffracted at 3.0 Å resolution (S4 Table). In this complex, GABARAP adopts the canonical Atg8 fold (Fig 6A), similar to what has been observed in previously reported structures of fusion proteins containing GABARAP and other LIR motifs [38–43]. In the structure of the complex, the predominant interactions at the binding interface with GABARAP involve the side chains of F456 and L459 of MARV NP-LIR, which are the residues in the first and fourth position of the LIR motif (Fig 6B). These residues insert respectively into hydrophobic pocket 1 (HP1) and hydrophobic pocket 2 (HP2) on the surface of GABARAP, which is consistent with structures of other canonical LIR motifs in complex with GABARAP [38–44]. At the binding interface, the aromatic ring of F456 of the MARV NP-LIR forms hydrophobic interactions with the side chains of Y5 (6.7Å), I21 (4.1Å), P30 (3.7Å), I32 (5.7Å), L50 (5.0Å) and F104 (3.9Å) as well as anion-π and cation-π interaction with E17 (3.4Å) and K48 (4.3Å) in HP1 of GABARAP, respectively (Fig 6B). In addition to these interactions involving F456, the side chain of L459 of MARV NP-LIR forms hydrophobic contacts with the side chains of Y49 (4.2Å), V51 (3.7Å), P52 (4.8Å), L55 (5.2Å), F60 (4.2Å) and L63 (3.5Å) in HP2 of GABARAP (Fig 6B). The binding interface with GABARAP is also stabilized by supplemental interactions, with residues both within and after the core four residues of the MARV NP-LIR motif. The additional interactions include hydrophobic interactions between the side chain of V457 from MARV NP-LIR and the side chains of Y49 (3.9 Å) and L50 (3.8Å) from GABARAP, as well as a potential hydrogen bond between the side chain of N460 from MARV NP-LIR and the side chain of R28 (4.2 Å) from GABARAP.

### MARV NP suppresses autophagy through F456 within its LIR motif

**Fig 7:**
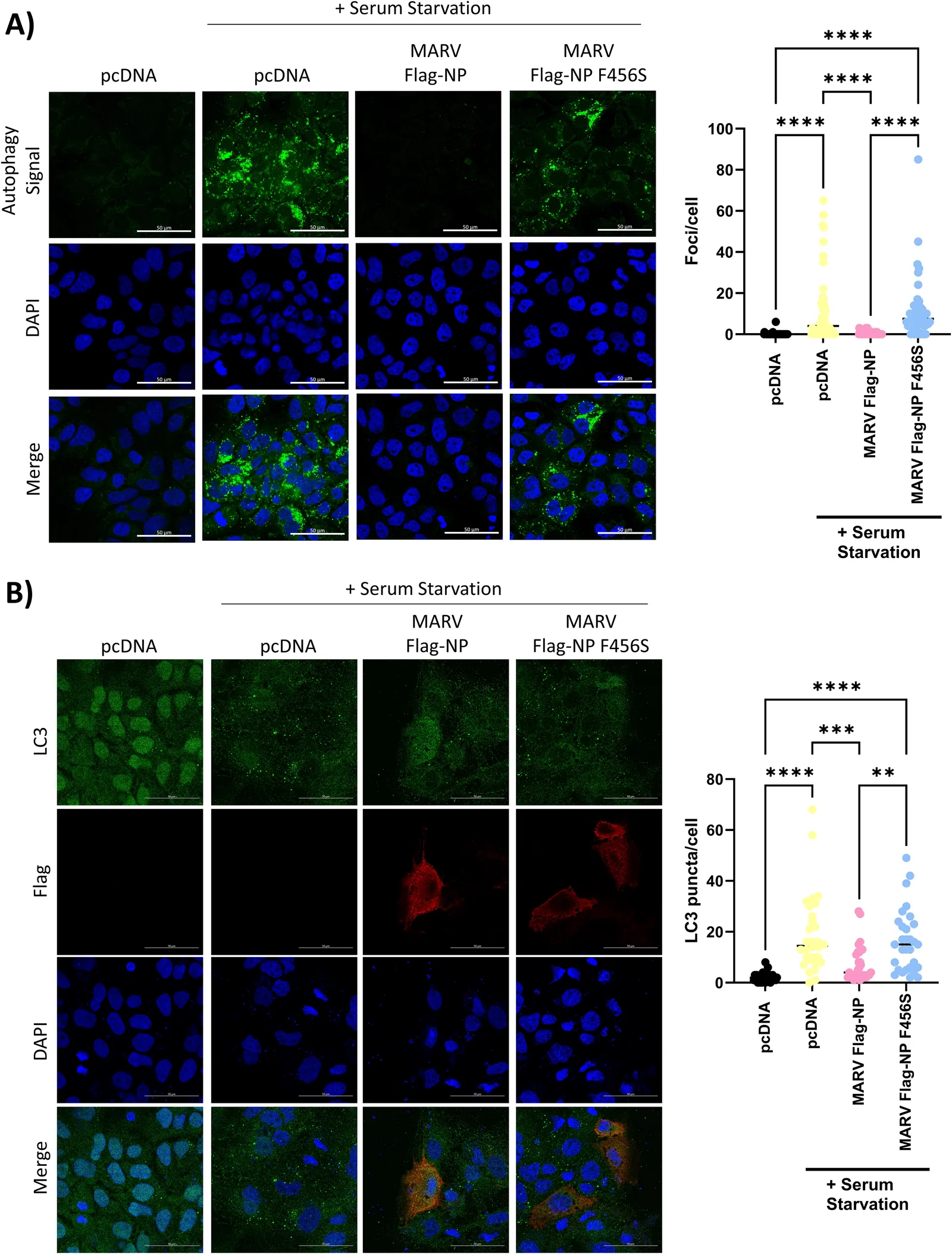
Suppression of autophagy by MARV NP requires F456 in the LIR motif. A) Huh-7 cells were subjected to the Abcam Autophagy Detection Kit Protocol, followed by confocal microscopy imaging. Green signal indicates the formation of autophagic vesicle. DAPI (blue) served as the nuclear marker. Cells transfected with pcDNA in complete media served as negative controls. Mock cells transfected only with empty vector pcDNA that were serum starved for 2 h served as the positive controls for autophagy. pcDNA, Flag tagged MARV NP, or Flag tagged MARV NP F456S transfected cells were subjected to serum starvation 2 h prior to collection. The number of foci per cell (50 cells/condition) are shown on the right panel. B) Cells were transfected and serum starved as described in panel A. At 24 hours post-transfection cells were collected and stained for LC3, Flag, and DAPI (nuclear), followed by confocal microscopy imaging. The number of LC3 foci per cell (30 cells/condition) are shown on the right panel. Statistical analysis was conducted using a one-way ANOVA with Tukey’s multiple comparison post hoc test ** = P ≤ 0.01, *** = P ≤ 0.001, **** = P ≤ 0.0001..

To evaluate the role of F456 in MARV NP-mediated modulation of autophagy, a 488-nm-excitable green fluorescent dye that selectively labels autophagic vacuoles was utilized. Huh7 cells were transfected with pcDNA, MARV Flag-NP, or MARV Flag-NP F456S. To initiate autophagy, cells were subjected to serum starvation for 2 h prior to collection at 24 hpt. Non-starved pcDNA transfected cells were included as a negative control and as expected exhibited very few cytoplasmic autophagic foci (Fig 7A). Conversely, pcDNA transfected cells that were serum starved displayed a significant increase in the number of autophagic foci per cell. Cells that were transfected with a Flag-tagged MARV-NP had a significantly lower number of autophagic foci as compared to the pcDNA transfected cells that were serum starved. This decrease in the number of autophagic foci was not observed in cells transfected with MARV Flag-NP F456S, which showed similar foci per cell to the pcDNA transfected cells under serum starvation.

During autophagosome formation, Atg8/LC3 proteins are conjugated to phosphatidylethanolamine (PE), allowing their stable incorporation into both the inner and outer membranes of the autophagosome [20, 21]. Given this, LC3 foci were assessed as another marker for autophagy. In non-serum-starved, pcDNA-transfected cells, LC3 was predominantly localized to the nucleus (Fig 7B), consistent with previous reports demonstrating that LC3 is maintained as a nuclear pool under homeostatic conditions [16]. Upon serum starvation in pcDNA transfected cells, LC3 was found in distinct foci observed in the cytoplasm. Cells expressing MARV Flag-NP under serum starvation conditions had a significantly reduced number of LC3 foci as compared to the pcDNA transfected cells that were serum starved. However, cells expressing MARV Flag-NP F456S under serum starvation conditions had a similar number of LC3 foci as serum starved pcDNA transfected cells. Collectively, these results support that MARV NP suppresses autophagy and this suppression is dependent on F456 within its LIR motif.

## Discussion

HFVs are comprised of a large array of viral families causing similar severe vascular manifestations including hemorrhaging, bruising, and edema [1, 45, 46]. HFVs, as well as other HVVs such as NiV and HeV, are difficult to study due to the large majority of these pathogens having a BSL-3 or BSL-4 laboratory requirement, [47, 48]. As a result, there is often limited research on the basic biology of HVVs which is necessary for vaccine and therapeutic development. Identifying interactions between HVV proteins and host proteins is crucial to understanding the progression of infections in humans, and a key focus area for pandemic preparedness. Here we developed the LIR Discovery Pipeline (LIR-DP) to help address this gap and enable the identification of a functional SLiM in viral proteins. The LIR-DP was inspired by our recent work demonstrating that RVFV NSs interacts with Atg8/LC3-family proteins through an LIR motif located in the C-terminus of the protein and this interaction suppresses autophagy in cells infected with RVFV [10]. In this work, we expand on these findings to integrate bioinformatics, AI, AlphaFold3, *in vitro* studies, and *in cellulo* validation to identify functional LIR motifs within viral proteins from 22 different HVVs. Using the LIR Discovery Pipeline, we identified 16 potential functional LIR motifs located within 18 different HVV proteins and validated three proteins experimentally as functional LIR motifs within HVV proteins including the MARV NP, the EBOV VP35, and the NiV P.

The ready availability of AlphaFold2 [49], and more recently AlphaFold3 [50], has served as the motivating factor to develop the LIR-DP as well as for other groups to develop similar tools for computational prediction of either LIR motifs specifically or SLiMs more generally. Most notably, there was a recent report of an AlphaFold SLiM screen for LC3-LIR interactions in autophagy [51]. In this report, it was observed that both AlphaFold2 and AlphaFold3 often fail to predict interactions of SLiMs in complex with their target partner in the context of the full-length proteins. This motivated our respective groups to develop fragment-based approaches, wherein the SLiM-containing protein was modeled as peptide segments of varying length in AlphaFold to model interactions with Atg8/LC3-family proteins. Specifically, *Stuke et al*. incorporated a sliding window algorithm that systematically creates overlapping peptide fragments of varying length and measures the resulting AlphaFold quality scores of their complex with Atg8/LC3-family proteins. They found that the shortest peptide fragments (*<*15 residues), for which AlphaFold2 typically fails to generate a multiple sequence alignment (MSA), produced the highest-scoring canonical Atg8/LC3-LIR complexes. We came to a similar conclusion in developing the LIR-DP. Short peptide fragments (16 residues in our case) yielded the highest reliable results with AlphaFold3, but our approach differs in notable ways. The HVV-focused analysis used front-end disorder filtering via IUPred3 to prioritize LIR candidates prior to performing the AlphaFold3 analysis to further verify that the LIR motif was located in a disordered region of the protein based on the pLDDT score (<50). This approach does sacrifice some automation for structural biology interpretation, including review of the putative LIR in the context of the often-predicted protein structure of HVV proteins. However, we also extended the AlphaFold analysis with post hoc FoldX energy calculations for more comprehensive biophysical analysis to boost confidence in modeling simulations prior to committing resources for biochemical and cell-based validation studies. By comparison, the *Stuke et al*. method offers the advantage of automating LIR discovery, including the possible identification of non-canonical LIR motifs, which may be useful for larger scale coarse-grained studies. However, there were no experimental validation to support the utility of the method to identify functional LIR motifs within biological systems.

To put our LIR-DP in even broader context as a general computational platform for analyzing for the presence of functional SLiMs present in a protein sequence, it can also be compared to the work of *Veinstein et al.* who developed an AlphaFold-based approach for predicting SLiM-mediated interactions [52] via ColabFold [53]. In this work, AlphaFold2 and AlphaFold3 were used to examine a structure independent benchmark of 26 interactions absent from representative structures in the PDB database. Their results from comparing different methods indicate that MiniPAE [54] was the best suited AlphaFold metric for screening for the presence of a SLiM. This study also demonstrated a comprehensive examination of other SLiM screening methods and metrics, and based on their benchmarked dataset, it seems to have a more general application to any type of SLiM, whereas the LIR-DP platform is currently specific for one SLiM, the LIR motif. However, this study did not report any results for LIR motifs, hence a direct comparison of the performance with our LIR-DP platform is not possible.

One key aspect to this work was that several of the predicted functional LIR motifs identified using the LIR-DP platform were validated experimentally with *in vitro* and *in cellulo* studies including the LIR motifs identified within the NiV P and the EBOV VP35. Interestingly, NiV P, EBOV VP35, and the previously identified LIR containing protein, RVFV NSs, are all virulence factors that modulate innate immune signaling and are interferon antagonists [55–58]. This is of particular interest as autophagy is often used by the immune system to degrade viruses and viral particles and conversely viruses have evolved mechanisms to prevent antiviral autophagy [59, 60]. Moreover, there is crosstalk between autophagy and interferon signaling, where interferon signaling can stimulate autophagy contributing to viral clearance [61]. Conversely autophagy can limit interferon expression through multiple mechanisms including degrading innate immune sensors such as cGAS-STING [62], degrading viral pathogen associated molecular patterns (PAMPs) [61], or through mediating the degradation of the interferon α/β receptor [61]. Based on prior studies showing RVFV NSs is both an interferon and autophagy antagonist [10, 58, 63], we speculate that NiV P and EBOV VP35 have evolved to dampen innate immune responses through interferon antagonism as well as the regulation of autophagy.

The MARV NP contained the third LIR motif identified with LIR-DP that was validated experimentally as being functional. Of the three LIR motifs validated, the MARV NP-LIR had the highest binding affinity for both GABARAP and LC3 in the ITC experiments and our structural studies demonstrated that the LIR bound to GABARAP in a canonical manner. In a previous study, the host autophagy chaperone BAG3 was shown to interact with the MARV VP40 matrix protein and this interaction inhibited budding of VP40 virus-like particles [64]. Given the antiviral role of autophagy during MARV infections, we speculate that the interaction of MARV NP with the Atg8/LC3-family proteins functions as a mechanism to inhibit autophagy and facilitate viral replication. Our data support this hypothesis as MARV NP inhibited autophagy and LC3 puncta formation in serum starved cells. Very few MARV NP host interacting partners have been identified; however, Tumor Susceptibility Gene 101 (TSG101), a member of the endosomal sorting complex, interacts with MARV NP to facilitate viral budding [65]. Interestingly TSG101 was found to facilitate the creation of amphisomes, intermediate organelles produced through the fusion of endosomes with autophagosomes, leading to increased autophagic flux [66]. Given this, it is possible that the interaction between MARV NP and TSG101, which facilitates viral replication, reduces the pool of TSG101 available for amphisome formation, thereby contributing to decreased autophagy. However, future studies are needed to clarify the impact of the interaction of the MARV NP with the Atg8/LC3-family proteins in virally infected cells.

While Atg8/LC3 proteins are heavily studied in the context of canonical autophagy, they are more broadly involved in the process of Atg8ylation. Atg8ylation is a recent concept, defined as the conjugation of Atg8/LC3 proteins to membranes and has been proposed as being similar to the ubiquitination of proteins [18, 19]. While Atg8ylation can occur during autophagy, there is emerging evidence of Atg8ylation of other cellular membranes other than autophagosomes. Atg8ylation is critical for membrane remodeling during conditions of homeostasis as well as during cellular stress including during viral infection [18, 19]. For example, HIV-1 infection leads to increased LC3B levels at the plasma membrane and Atg8ylation was found to facilitate viral entry independent of autophagy [67]. Given this, it is intriguing to consider the HVV proteins containing LIR motifs in the broader context of Atg8ylation. While not validated, we identified EBOV GP as containing a putative LIR. This is of interest considering that LC3B was found in macropinocytic vesicles and shown to be required for trafficking of EBOV into the cell via macropinocytosis [68]. Based on this we speculate that the putative interaction between EBOV GP and LC3B may facilitate viral entry. As a number of the candidate LIRs are found within HVV structural proteins, such as nucleoproteins that mediate virion formation and budding, future studies should consider these proteins in the broader context of Atg8ylation.

The present study demonstrates the versatility of the LIR-DP platform for identifying HVV-host protein interactions involving LIR motifs. Although only a handful of proteins were validated in cells, future work will focus on the importance of these interactions for autophagy and viral replication. In addition, the testing of two of the putative LIR motifs (DENV CP and GTOV Z) indicated that these were not functional *in vitro* or *in cellulo*. The most probably explanation of their lack of functionality was the abundance of positively charged amino acids residues within the region of the LIR motifs as indicated by the high pI of the LIR-containing peptides tested. It is well documented that negatively charged amino acids in the vicinity of LIR motifs, including phosphorylated amino acids, often enhance the strength of the interaction between Atg8/LC3-family proteins and LIR motifs [22, 37]. This characteristic is also found in several other SLiM motifs binding to their target proteins including SUMO-interacting motifs (SIMs) binding to SUMO-family proteins [69–71] and acidic transactivation domains (TADs) binding to transcription regulatory factors [72, 73]. In all three cases, the target proteins that these SLiMs interact with have an electropositive binding surface. Taken together, this suggests that the LIR-DP pipeline has the potential to be expanded to include additional criteria for identifying a function SLiM such as pI in the case of the LIR motifs, SIMs and acidic TADs. In conclusion, this work demonstrates the utility of the LIR-DP for identifying functional LIR motifs in viral proteins but could also be used to identify host protein interactions with Atg8/LC3-family proteins. In additions, it is versatile enough to be adapted for use with other types of SLiMs implicated in host-viral or host-host interactions through the incorporation of some simple adjustments to the pipeline.

## Materials and methods

### Initial LIR motif identification

HVV protein sequences were analyzed using the eukaryotic linear motif (ELM) and iLIR databases to identify LIR motifs based on pattern recognition. We scanned proteins that contain the primary human relevant LIR motif (LIG_LIR_GEN_1) for further analysis. LIG_LIR_GEN_1 (http://elm.eu.org/elms/LIG_LIR_Gen_1) considers up to 2 residues before and 8 residues after the core 4 residue sequence. Proteins containing non-human LIR variants (ELM identifier LIG_LIR_NEM_3 and LIG_LIR_APIC_2) were excluded. We also scanned for the LC3C specific variant (ELM identifier LIG_LIR_LC3C) which only returned a single possible interaction that was not considered further. Proteins containing a candidate LIR were next analyzed using IUPred3 [33] to estimate the overall disorder of the region of the protein where the LIR is located in the protein sequence. LIRs located within a region with an average disorder score over above 30 were selected to continue to the next step in the pipeline.

### Alpha-fold analysis

AlphaFold3 (AF3) version 3.01 pre-trained multimer models and parameters from January 27, 2025 were used to model structures of complexes between the selected LIR motifs (Table 1) in either the LC3A or GABARAP proteins [50]. Each of the pre-trained AlphaFold calculations were run using random seeds creating bound models of LIR motif-containing peptides complexed with LC3A and GABARAP. FoldX version 5.1 was then applied to the resulting models to refine the positions and calculate energies of the peptide complexes with LC3A and GABARAP [74]. A series of biophysical parameters calculated from each structure were used to evaluate their overall quality including total energy in kcal/mol, and accessible surface area of the LIR motif before and after binding. These parameters allowed for ranking the interactions of the models based on binding specificity and energy. Similarly, the resulting models were inspected using UCSF Chimera to verify the binding mode of the LIR motifs to either LC3A or GABARAP and to compare with other available protein structures from the RCSB Protein Data Bank that contain an LIR complexed to either LC3A or GABARAP.

### Peptides and protein expression and purification

The sequences encoding for LC3A (residues 1–121 of human LC3A), GABARAP (residues 1–117 of human GABARAP), MARV NP-LIR-GABARAP (residues 454–463 of MARV NP fused to the N-terminal of residues 1–117 of GABARAP with a two glycine residue separation), RVFV NSs-LIR (residues 256–265 of RVFV NSs), MARV NP-LIR (residues 448–469 of MARV NP), NiV P/V/W-LIR (residues 45–66 of NiV P), GTOV Z-LIR (residues 2–21 of GTOV Z), EBOV VP35-LIR (Residues 75–93 EBOV VP35) and DENV CP-LIR (residues 2–23 of DENV CP) were synthesized as oligonucleotides (Integrated DNA Technologies) with flanking BamHI and EcoRI restriction enzyme sites and cloned into a modified pGEX-2T vector (Amersham) with a Tobacco Etch Virus (TEV) protease cleavage site replacing the original thrombin cut site. In the case of the MARV NP-LIR, the DENV CP-LIR, EBOV VP35-LIR and the NiV P/V/W-LIR a Tyrosine residues was added at the C-terminal end of the peptide to allow for quantifying the peptide concentration by UV absorbance at 280 nm. The proteins were expressed as GST-fusion proteins in E. coli host strain TOPP2. Cells were grown in Luria Broth (LB) medium supplemented with 100 mg/mL ampicillin overnight at 37°C. The next day, cells were diluted one part in four in LB plus antibiotics. Protein expression was induced with 1 mM IsoPropyl-b-D-ThioGalactopyranoside (IPTG; Inalco) for 4h at 30°C. Cells were pelleted by centrifugation (15min, 12,000 x g) and resuspended in lysis buffer (20 mM Tris-HCl pH 7.4, 1M NaCl, 0.2 mM EDTA, 1 mM DTT) and lysed using a French Press. The resulting suspension was centrifuged (1h, 105,000 x g, 4°C) and the supernatant incubated (1h, 4°C) with a Glutathione Sepharose 4B (GSH; Cytiva) resin. The resin was then rinsed three times with TEV buffer (20 mM NaH2PO4/Na2HPO4 pH 7.4, 125 mM NaCl, 5 mM DTT) and incubated overnight at room temperature with the TEV protease. The next day, the proteins were further purified by ion exchange chromatography using either a High-Performance SP-Sepharose (Cytiva) column (LC3A, LC3B, GABARAP, GTOV Z-LIR and DENV CP-LIR) or a High-Performance Q-Sepharose (Cytiva) column (RVFV NSs-LIR, MARV NP-LIR, NiV P/V/W-LIR, EBOV VP35-LIR). For the ion-exchange chromatography, the columns were equilibrated in 20 mM sodium phosphate buffer pH 6.5 with 1 mM DTT (buffer A) and the proteins eluted with a gradient of 20 mM sodium phosphate buffer pH 6.5 with1 M NaCl and 1 mM DTT (buffer B). The fractions containing the protein from the ion exchange columns were then dialyzed into 20 mM sodium phosphate buffer pH 6.5 with 50 mM NaCl and 1 mM DTT and purified by gel filtration chromatography over a Sepharose 12 10/300GL column (GE Healthcare) and the final purified proteins stored at -80°C prior to usage in ITC and crystallography experiments.

### ITC experiments

Proteins were dialyzed overnight at room temperature into 20 mM sodium phosphate buffer pH 6.5 with 50 mM NaCl. Protein concentrations were determined by UV absorbance at 280 nm. ITC measurements were performed at 25°C using a VP-ITC calorimeter (MicroCal). Data were analyzed using Origin Software and all experiments fit the single binding site model with 1:1 stoichiometry. Standard deviations in *K*_D_ values were determined from duplicate measurements or more.

### Crystallization and data collection

The MARV NP-LIR-GABARAP fusion protein was dialyzed into 25 mM MES, 50 mM NaCl with 1mM DTT buffer and used at concentrations varying between 150–700 μM. The crystals were generated using the hanging drop vapor diffusion method. The selected crystals following screening of varying conditions were cryoprotected in a mother liquor containing 20% glycerol. Diffraction data collected using Pilatus3S 6M at beamline 08-ID of the Canadian Light Source (CLS) or either a Pilatus3S 6M detector or at the beamline IDB7 of the Macromolecular Cornell High Energy Synchrotron Source (MacCHESS). Datasets were indexed, integrated, and scaled using HKL2000 (HKL Research, Inc.).

### Structure determination and refinement

For calculation of the structures from the crystal data, the initial phases were generated by molecular replacement using the crystal structure of GABARAP (PDB:1GNU) as a search template. The final phases were determined following iterative cycles of model building with Coot and refinement using PHENIX [75]. Test data sets were selected randomly from the observed reflections prior to refinement. Statistics for the final models were obtained with PHENIX and Molprobity, and the figures were prepared with PyMOL [76].

### Cell culture

Human embryonic kidney cells (HEK293T) were acquired from American Type Culture Collection (ATCC, CRL-3216) and were maintained in Dulbecco’s modified minimum essential medium (DMEM) supplemented with 10% fetal bovine serum (FBS) and 1% L-glutamine. Human hepatocellular carcinoma cells (Huh-7), were maintained in Dulbecco’s modified minimum essential medium supplemented with 10% FBS, 1% penicillin/streptomycin, 1% L-glutamine, 1% nonessential amino acids, and 1% sodium pyruvate [77]. All cell lines were maintained at 37°C with 5% CO_2_.

### Antibodies

The antibody for LC3B (3868) was obtained from Cell Signaling Technology (Beverley, MD). Cross reactivity of this antibody with LC3A may occur and thus all blots are labeled as “LC3.” The Anti-Flag (F1804) specific monoclonal antibody was obtained from Millipore Sigma (Rockville, MD). Antibodies specific for HRP Anti-beta actin (ab4900) and Green fluorescent protein (GFP) (ab290) were obtained from Abcam (Waltham, Boston). Goat anti-mouse IgG (32430) and goat anti-rabbit IgG (32460) secondary antibodies were obtained from ThermoFisher Scientific (Rockford, IL).

### Expression plasmids

For expression of GFP-GABARAP in cells, the sequence encoding human GABARAP was inserted into the pEGFP-C1 vector using BglII/EcoRI restriction sites and subsequently subcloned into the pCDNA3.1 vector. Vector pCAGGS containing the Marburg virus, Musoke isolate (GenBank: DQ217792) Nucleoprotein Gene with N-Terminal Flag Tag (Cat. # NR-49344) (MARV Flag-NP), vector pCAGGS containing the Ebola virus, SLE/2014/Makona-G3848 isolate (GenBank: KM233110) Nucleoprotein Gene with N-Terminal Flag Tag (Cat. # NR-51474) (EBOV Flag-NP), and vector pCAGGS containing the Ebola virus, Mayinga isolate (GenBank: AF086833.2) VP35 Gene with N-Terminal Flag Tag (Cat. # NR-49387) (EBOV Flag-VP35) were obtained through BEI Resources, NIAID, NIH. pCAGGS MARV Flag-NP F454S, was created through a double nucleotide change of TTT (nucleotide positions: 5621, 5622, 5623) (phenylalanine) to TCA (serine). pCAGGS EBOV Flag-VP35 F82S, was created through a double nucleotide change of TTT (nucleotide positions: 4616, 4617, 4618) (phenylalanine) to TCA (serine). The pcDNA 3.1 (+) vector expressing the NiV isolate 810428 (GenBank: MK673566.1) P with a C-terminal Flag Tag (NiV P-Flag), was synthesized by Epoch Life Sciences. pcDNA 3.1 (+) NiV P F55S-Flag was created through a double nucleotide change of TTT (nucleotide positions: 1070, 1071, 1072) (phenylalanine) to TCA (serine). pcDNA 3.1 (+) vectors expressing the GTOV strain AV 97021119 (GenBank: AY562389.1) Z with a C-terminal Flag Tag (GTOV Z-Flag) or the DENV type 1 (NCBI Reference Sequence: NC_001477.1) CP with a C-terminal Flag Tag (DENV CP-Flag) were also synthesized by Epoch Life Sciences.

### Transfections

HEK293T or Huh-7 cells were transfected with viral protein expression plasmids or empty vector pcDNA plasmids (mock). For experiments examining GABARAP interactions, HEK293T or Huh-7 cells were transfected only with GFP plasmid (mock) or co-transfected with GFP-GABARAP protein and viral expression plasmids. All transfections were performed using TransIT-293 transfection reagent (Mirus, 2704) according to the manufacturer’s instructions. Briefly, 19 μL plasmid DNA (of a 1mg/mL stock), 1.9 mL Opti-MEM I Reduced-Serum Medium, and 57 μL room-temperature warmed TransIT-293 Reagent was added to microcentrifuge tubes and mixed, then incubated for 30 min at room temperature. HEK293T or Huh-7 cells (seeded at 2.1x10^6^ (HEK293T) or 4x10^6^ (Huh-7) per 75 cm2 flask) were transfected with TransIT-293 reagent DNA complex via adding the mixture dropwise and rocking to distribute the plasmids.

### Immunoprecipitation

Cells underwent trypsinization (Corning, 25–053-CI), were resuspended in cell culture media, and centrifuged at 2,500 rpm for 10 min. The supernatant was discarded, and pellets were resuspended in 1 mL of sterile PBS (ThermoFisher, 10010023), followed by centrifugation at 2,500 rpm for 10 min. After discarding the PBS, pellets were vigorously resuspended in clear lysis buffer (50 mM Tris-HCl, pH 7.4; 120 mM NaCl; 5 mM EDTA; 0.5% NP-40; 50 mM NaF; 0.2 mM Na_3_VO_4_; and EDTA-free complete protease inhibitor cocktail [Roche, 11697498001]) based on flask size (300 μL per 75 cm² flask)). Samples were incubated on ice and vortexed every 5 min for 20 min. Following incubation, samples were centrifuged at 15,000 rpm for 10 min. Pellets were discarded, and lysates were quantified using Bradford reagent (ThermoFisher, 23236). One mg of protein was used for each immunoprecipitation (IP) sample; the sample volume was balanced with clear lysis buffer for a total sample volume of 250 μL. Input samples were collected as 2% of the IP sample volume, except for NiV P experiments where 1% input samples were collected. IP samples were incubated with 1 μg of LC3 antibody or GFP antibody and samples were incubated overnight rotating at 4°C. The next day, Protein G Dynabeads (Invitrogen, 10003D) were prepared by obtaining 50 μL beads per sample, washing twice with citrate phosphate buffer (0.5M, pH 5.0; 1L DI water, 18.15g of Sodium Phosphate Dibasic Dihydrate, 9.605 g of Citric Acid), twice with PBS, and resuspension in PBS (50 μL per sample). Fifty μL of the prepared Protein G beads was added to each IP sample and they were incubated at room temperature rocking for 45 min. After the rotation, samples were washed with 500 μL of TNE150 (50 nM Tris-HCl (pH 7.5), 150 mM NaCl, 1 mM EDTA, and 0.1% NP-40), 500 μL of TNE 50 (50 nM Tris (pH 7.5), 150 mM NaCl, 1 mM EDTA, and 0.1% NP-40), and 250 μL of PBS. All washes were discarded and samples were eluted in blue lysis buffer (25 μL per IP sample and 1:1 ratio for input samples). Blue lysis buffer is composed of 25 mL 2x Novex Tris-Glycine Sample Loading Buffer SDS (Invitrogen, LC2676), 20 mL T-PER Tissue Protein Extraction Reagent (Thermo Scientific, 78510), 200 μL 0.5 M EDTA pH 8.0, EDTA-free complete protease inhibitor cocktail (Roche, 11697498001), 80 μL 0.1 M Na3VO4, 400 μL 0.1M NaF, and 1.3 mL 1M DTT. Then samples underwent western blot protocol.

### Western blot

Samples were collected and prepared as described in the immunoprecipitation protocol. Proteins were separated on 4–12% Bis-Tris gels, wet transferred onto PVDF membranes (VWR, 490007) for 1h, and blocked at room temperature for 1h with 5% bovine serum albumin (BSA) (ThermoFisher, B14) in Tris-buffered saline containing 0.1% Tween-20 (TBST). Membranes were probed with primary anti-Flag, anti-LC3B, or anti-GFP antibodies, which were diluted in 5% BSA blocking buffer and incubated overnight on a rocker at 4°C. The membranes were washed with tris buffered saline TBS, TBST, TBS for 5 min each, incubated for 1h at room temperature rocking with secondary anti-rabbit (LC3, GFP) or secondary anti-mouse (Flag) diluted in blocking buffer (1:1000), then washed with TBS, TBST, TBST, TBS for 5 min each. The membranes were imaged via chemiluminescence using the SuperSignal West Femto Maximum Sensitivity substrate kit (Thermo Scientific, 34095) on the Bio Rad ChemiDoc MP Imaging System.

### Western blot quantification

Quantification of western blot images were performed with Fiji Image J Software. Raw, uncompressed files were imported into Fiji Image J. Rectangular regions of interest (ROIs) of identical size were created around each individual Flag-tagged protein bands, LC3 band, and adjacent background regions. Mean pixel density values were recorded for all bands and background. In ImageJ, pixel density is reported inversely proportional to signal intensity, thus all values were inverted prior to analysis using the formula 255-(mean pixel density value). For each Flag and LC3 band, net signal intensity was then calculated by subtracting the inverted background values from the inverted band values. For each lane, Flag protein levels were normalized by calculating the ratio of Flag band intensity to LC3 band intensity. Then, normalized IP values were further normalized to the input values to account for differences in protein expression. Final normalized values were expressed relative to the WT HVV proteins by setting these proteins to 100%, and protein values were expressed relative to WT, to properly reflect differences in binding. All quantification was performed as described with identical analysis across all IP experiments.

### Autophagy assay

Huh7 cells were seeded on coverslips in six-well plates (4x10^5^ cells per well) and grown to 70% confluency. Cells were then washed with PBS three times and transfected with 2.5 µg of pcDNA, plasmids expressing flag tagged MARV NP, or MARV NP F456S using the TransIT-293 transfection reagent (Mirus, 2704) according to the manufacturer’s instructions. Twenty-two hours post-transfection cells were serum starved for 2 h. To accomplish this, media was removed, and cells were washed with PBX two times. Serum starvation media (1% BSA, 140 mM NaCl, 1mM CaCl2, 1mM MgCl2, 5mM glucose, and 20 mM HEPES [pH 7.4]) was added to cells and cells incubated for 2 h prior to collection. pcDNA transfected cells that were not subjected to serum starvation were included as a control. Cells were subjected to the Abcam Autophagy Detection Kit Protocol (Abcam, ab139484) according to the manufacturer’s instructions, followed by imaging via confocal microscopy. Slides were imaged using a water-immersion 60 × objective lens on a Zeiss LSM 880 confocal laser scanning microscope. The number of foci per cell were counted for 50 cells/condition manually, assisted by ImageJ FIJI Software, with a representative image shown.

### Confocal microscopy

Huh7 cells were seeded on coverslips in six-well plates (4x10^5^ cells per well) and grown to 70% confluency. Cells were then washed with PBS three times and transfected with 2.5 µg of pcDNA, plasmids expressing Flag tagged MARV NP or MARV Flag-NP F456S using the TransIT-293 transfection reagent (Mirus, 2704) according to the manufacturer’s instructions. Twenty-two hours post-transfection cells were serum starved for 2 h as described above. At the time of collection, cells were washed with PBS (−Ca^2+^ and −Mg^2+^) and then fixed with 4% (wt/vol) neutral buffered paraformaldehyde. Cells were permeabilized with 0.1% (vol/vol) Triton X-100 in PBS for 10 min followed by washing three times with PBS. Cells were blocked in 1% bovine serum albumin and 0.025% Triton X-100 in PBS, washed three times with PBS. The cells were probed overnight with anti-Flag antibody (1:1000) and anti-LC3B antibody (1:100). Next, cells were washed three times with 0.025% Triton X-100 in PBS and probed with Alexa Fluor 568 goat anti-mouse secondary antibody (1:1000; Invitrogen, 11004) and Alexa Fluor 488 goat anti-rabbit secondary antibody (1:1000; Invitrogen, 11008). Nuclei were counterstained with DAPI (4,’6-diamidino-2-phenylindole, 1:1000). Coverslips were mounted to glass slides using Fluoromount-G (Southern Biotech, 0100). Slides were imaged using a water-immersion 60 × objective lens on a Zeiss LSM 880 confocal laser scanning microscope. Samples were imaged at least 10 times each across 2 biological replicates. LC3 puncta was quantified for 30 cells/condition manually across the 2 biological replicates, assisted by ImageJ FIJI Software, with a representative image shown. For cells transfected with Flag-tagged MARV NP or Flag-tagged MARV NP F456S, LC3 puncta quantification was restricted to cells positive for viral protein expression.

### Statistics

Statistical analysis was conducted using a one-way ANOVA and post-hoc Sidak’s test unless otherwise noted via GraphPad Prism Software. ns = not significant (P > 0.05), * = P ≤ 0.05, ** = P ≤ 0.01, *** = P ≤ 0.001, **** = P ≤ 0.0001.

## Supporting information

S1 Fig AlphaFold3 and pLDDT analysis of MARV NP and Lassa virus NP. Marburg virus (MARV) nucleoprotein (NP) (panel A and zoomed in image in panel B) and Lassa virus (LASV) NP (panel C and zoomed in image in panel D) were analyzed via AlphaFold3 and predicted local distance difference test (pLDDT) performed.
(TIF)

S2 Fig GTOV Z and DENV CP do not interact with LC3. (A, B) HEK293T cells were transfected with the indicated plasmids and harvested 24 hpt. Co-immunoprecipitation was performed using anti-LC3 antibodies, followed by western blotting with anti-Flag and anti-LC3 antibodies.
(TIF)

S3 Fig NiV P does not interact with LC3 *in cellulo.* HEK293T cells were transfected with the indicated plasmids and harvested 24 hpt. Co-immunoprecipitation was performed using anti-LC3 antibodies, followed by western blotting with anti-Flag and anti-LC3 antibodies.
(TIF)

S4 Fig EBOV VP35 does not interact with GABARAP *in cellulo.* Huh7 cells were transfected with the indicated plasmids and harvested 24 hpt. Co-immunoprecipitation was performed using anti-GFP antibodies, followed by western blotting with anti-Flag and anti-GFP antibodies.
(TIF)

S1 Table List of 166 viral proteins curated from the proteomes of 22 highly virulent viruses (HVVs). Information provided includes the virus name, viral protein name, protein length, Uniprot protein ID, and Uniprot virus ID. Proteins in italics contains sequences captured within their corresponding polyprotein and were not independently run through the LIR-DP.
(XLSX)

S2 Table List of 43 putative LIR motifs from HVVs identified via sequence and IUPred3 disorder prediction. Information provided includes the virus name, protein LIR Sequence, LIR Start Amino Acid, LIR Stop Amino Acid, and the protein Uniprot ID.
(XLSX)

S3 Table Sequences and pI of peptides with putative LIR motifs. Peptides used for IHC are displayed along with their corresponding pI.
(XLSX)

S4 Table Data collection and refinement statistics for GABARAP-MARV-LIR complex. Values in parentheses are for highest-resolution shell. Rsym = ∑ hkl ∑i|Ihkl,i − < Ihkl > , where Ihkl,i is the intensity of an individual measurement of the reflection with Miller indices hkl and Ihkl is the mean intensity of the reflection. Rwork = ∑ hkl||Fo| − |Fc|| / ∑ hkl |Fo|, where |Fo| is the observed structure-factor amplitude and |Fc| is the calculated structure-factor amplitude. Rfree is the R factor based on at least 500 test reflections that were excluded from the refinements. CHESS (Cornell High Energy Synchrotron Source). a, Reflection for Fo > 0. b, MolProbity analysis.
(XLSX)

S5 Table Values used to build graphs in Figures 3, 4, and 5. Values shown are the percent relative binding, with the wildtype HVV set to 100%.
(XLSX)

S6 Table Values used to build graphs in Figure 7. Values shown for panel A are autophagic foci/cell and values shown for panel B are LC3 foci/cell. (XLSX)
